# Emerging patterns of fluoroquinolone resistance in *Campylobacter jejuni* in the UK [1998–2018]

**DOI:** 10.1099/mgen.0.000875

**Published:** 2022-09-26

**Authors:** Dessislava Veltcheva, Frances M. Colles, Margaret Varga, Martin C. J. Maiden, Michael B. Bonsall

**Affiliations:** ^1^​ Department of Biology, University of Oxford, Oxford, UK

**Keywords:** *Campylobacter jejuni*, fluoroquinolones, antimicrobial resistance, statistical modelling, clonal complex, Multi-locus sequence typing, Generalised Linear Model

## Abstract

*
Campylobacter jejuni* (*C.jejuni)* is the most common causative agent of bacterial food poisoning worldwide and is known to be genetically highly diverse. *C. jejuni* is increasingly resistant to fluoroquinolone antibiotics, but very few studies have investigated variant-specific patterns of resistance across time. Here we use statistical modelling and clustering techniques to investigate patterns of fluoroquinolone resistance amongst 10,359 UK isolates from human disease sampled over 20 years. We observed six distinct patterns of fluoroquinolone sensitivity/resistance in *C. jejuni* across time, grouping by clonal complex (CC). Some CCs were fully resistant, some shifted from susceptible to resistant following a sigmoidal shape, and some remained susceptible over time. Our findings indicate that the fluoroquinolone resistance patterns of *C. jejuni* are complicated and cannot be analysed as a single species but divided into variant dynamics so that the factors driving resistance can be thoroughly investigated.

## Data Summary

All data used in this study are open access and can be found in the PubMLST database (https://pubmlst.org/organisms/campylobacter-jejunicoli/) by searching for public project No.110, ‘1998–2018 UK human isolates (n=10,359)’ on the *Campylobacter jejuni/coli* isolates collection public projects dropdown menu on the ‘isolate collection’ section, or by using the following link: https://pubmlst.org/bigsdb?db=pubmlst_campylobacter_isolates&page=query&project_list=110&submit=1 [1]. In addition, it can also be searched by publication in the PubMLST website.

**Impact Statement**

Antimicrobial resistance in *C. jejuni* poses a major clinical problem that could ultimately eliminate the efficacy of all known antibiotics in the future. Fluoroquinolone resistance is known to be increasing in *C. jejuni* over time. However, it is a diverse organism and patterns of resistance among its variants, known as clonal complexes, have not yet been well characterized. Our study uses statistical modelling, including generalized linear models and cluster analysis, to identify six distinct patterns of resistance. We estimate that two of the six patterns will reach near complete resistance levels by 2040. This study contributes new insights in the patterns of resistance among the diverse phylogeny of *C. jejuni* . In addition, we propose that antimicrobial resistance should be studied at the level of variants rather than species to describe the underlying traits affecting the evolution of resistance more accurately.

## Introduction

Campylobacteriosis is the most widely known cause of human gastroenteritis, affecting 400–500 million individuals worldwide annually [2]. In the UK, healthcare spending related to campylobacteriosis is approximately GBP £50 million per annum [3]. Approximately 90 % of campylobacteriosis cases are caused by the bacterium *Campylobacter jejuni *, and the rest are primarily caused by *Campylobacter coli* [4]. Approximately 600,000 people are infected annually in the UK alone and approximately 100 people are hospitalized each year [5, 6]

*Campylobacter* resides in the intestines of wild and domesticated animals, including chickens, cows, and pigs [7], all of which are potential sources of human infection. However, in high-income countries, contaminated chicken meat is the most common source of infection, causing an estimated 60–80 % of human disease in the UK [8]. Approximately 73 % of chickens sold in supermarkets are contaminated with *Campylobacter* and at least 7 % have contamination on the external packaging [9]. Incidence of campylobacteriosis is sporadic, but they have been known to lead to outbreaks in restaurants from food sources such as chicken liver pâté or undercooked chicken meats [10]. Usually campylobacteriosis is self-limiting and symptoms resolve within 1 or 2 weeks, but antibiotics such as macrolides can be prescribed if the condition is prolonged, or for vulnerable individuals, including the young, the immunocompromised and the elderly [11].

Fluoroquinolone resistance in *C. jejuni* is part of the World Health Organization (WHO) high-alert watch list and it is increasing, threatening global antibiotic efficacy [12, 13]. A single-point mutation in the C257T at the *gyrA* gene within the quinolone resistance-determining region (QRDR) leads to Thr86Ile amino acid substitution, which can cause phenotypic resistance to fluoroquinolone in *C. jejuni* [14–17]. This amino acid change can be maintained stably in *Campylobacter* populations without antibiotic selection pressure and *in vivo* chicken colonization studies have shown that fluoroquinolone-resistant *Campylobacter* mutants do not carry a fitness burden [18]. In addition, these resistant isolates have been found to outcompete susceptible isolates, indicating that the mutation confers a selective advantage even in the absence of the antibiotic pressure [18]. It has also been shown that despite discontinuing the use of fluoroquinolones, *Campylobacter* found in farms continue to have high fluoroquinolone resistance even after four years, which suggests that once campylobacters become resistant to fluoroquinolones, it is challenging to reduce their prevalence [12].

*C. jejuni* has high genetic diversity, which makes investigating changing patterns of resistance particularly challenging [19]. Multi-locus sequence typing (MLST) schemes have revolutionized classification of *C. jejuni* globally, with clonal complex (CC) designation based upon seven-locus MLST giving insight into sources of infection [20, 21]. Although an EU growth promoter ban came into place in 2006 for livestock, antibiotics continue to be permitted for therapeutic reasons. While some studies highlight the trend in resistance, the in-depth dynamics and trends of fluoroquinolone resistance within *C. jejuni* CCs following legislative changes in antibiotic use on farms have not been thoroughly studied to date [22, 23].

Previous work has investigated correlation of CC and antimicrobial resistance change across six years from 2003 to 2009 from isolates in Oxfordshire, UK. However, this work did not include other UK regions [24]. Other studies have compared antimicrobial resistance across time but, they only considered *Campylobacter* as a single species, rather than examining variant dynamics [23]. This work aimed to readdress this and investigate patterns of changes in fluoroquinolone resistance changes across *C. jejuni’s* clonal complexes.

### Gap statement/Aim

With increasing emphasis being placed on antibiotic stewardship in treating human infections, as well as livestock (including an EU ban on growth-promoting antibiotics in 2006), a decrease in fluoroquinolone resistance at a population level in recent years might be anticipated. Exploring variant-based levels of fluoroquinolone resistance in clinical isolates can provide insight into the sources of antimicrobial resistance emergence. This study aims to assess patterns in fluoroquinolone resistance in UK isolates over time, in relation to clonal complex.

## Methods

### Metadata acquisition

The data used in our study are publicly available from the PubMLST database [1]. A total of 10,359 *Campylobacter* isolates were obtained from the PubMLST database and used in this study. Our search criteria were: (‘Species= *Campylobacter jejuni *’ *AND* ‘Country=UK’ *AND* ‘1990>=Year<=2020’ *AND* ‘N50>=20,000’ *AND* ‘1.4Mb<=Genome Size <= 1.8 Mb’ *AND* ‘Contigs<=50’ AND ‘source=human_stool’). The samples used can also be found directly on PubMLST public project No.110 - ‘1998-2018 UK human isolates (n=10,359)’: https://pubmlst.org/bigsdb?db=pubmlst_campylobacter_isolates&page=query&project_list=110&submit=1.

### Bioinformatics pipeline and software usage

Using whole-genome sequence data for each of the isolates chosen from the PubMLST database, the *gyrase A* (*gyrA*) (CAMP0950) locus was queried. The *gyrA* nucleotide sequences for all isolates were aligned to one another using MAFFT/7.305 [25]. The nucleotide alignment was translated to an amino acid alignment using Transeq [26]. Due to the absence of phenotypic data for the majority of the samples, the presence or absence of the Thr86Ile amino acid substitution was used to infer resistance or susceptibility, respectively [24, 27]. There are other mutations that are also known to lead to fluoroquinolone resistance, such as Asp90Asn, Thr86Lys, Thr86Ala, Thr86Val and Asp90Tyr, but these usually occur at lower frequency [15, 17]. Therefore, we focused solely on the amino acid substitution of Thr86Ile in this study.

The provenance and phenotypic data from the data acquisition step and the 86^th^ position of GyrA were then aggregated for further analysis. The bootstrap analysis, heatmap clustering and GLM models were run using the R programming language [28], with libraries including ggplot2, stats, d3heatmap, reshape2, heatmap, dplyr, tidyverse, lme4 and Viridis. Code for the methods described is available here: https://github.com/bgrdessislava/Campyjejuni_gyrA.

### Identifying patterns in resistance over time

Due to varying sample sizes across years, bootstrap analysis was used to resample 100 isolates with replacement and compute the percentage resistance. This sampling was repeated 100 times, and a linear model was fitted to visualize the changes in resistance across time. To investigate the levels of resistance further, the amino acid of gyrase86, whether it was threonine or isoleucine, was used to cluster the CCs over time. To investigate the temporal dynamics, we used logistic regression through time to understand changes in the levels of resistance. Generalized linear models using a binary response variable (for susceptible or resistance variant) with year and group as covariates were used to investigate these trends across time further. Further code details can be found in GitHub (see above).

## Results

### *C. jejuni* fluoroquinolone resistance increased over time, 1998–2018

**Fig. 1.**
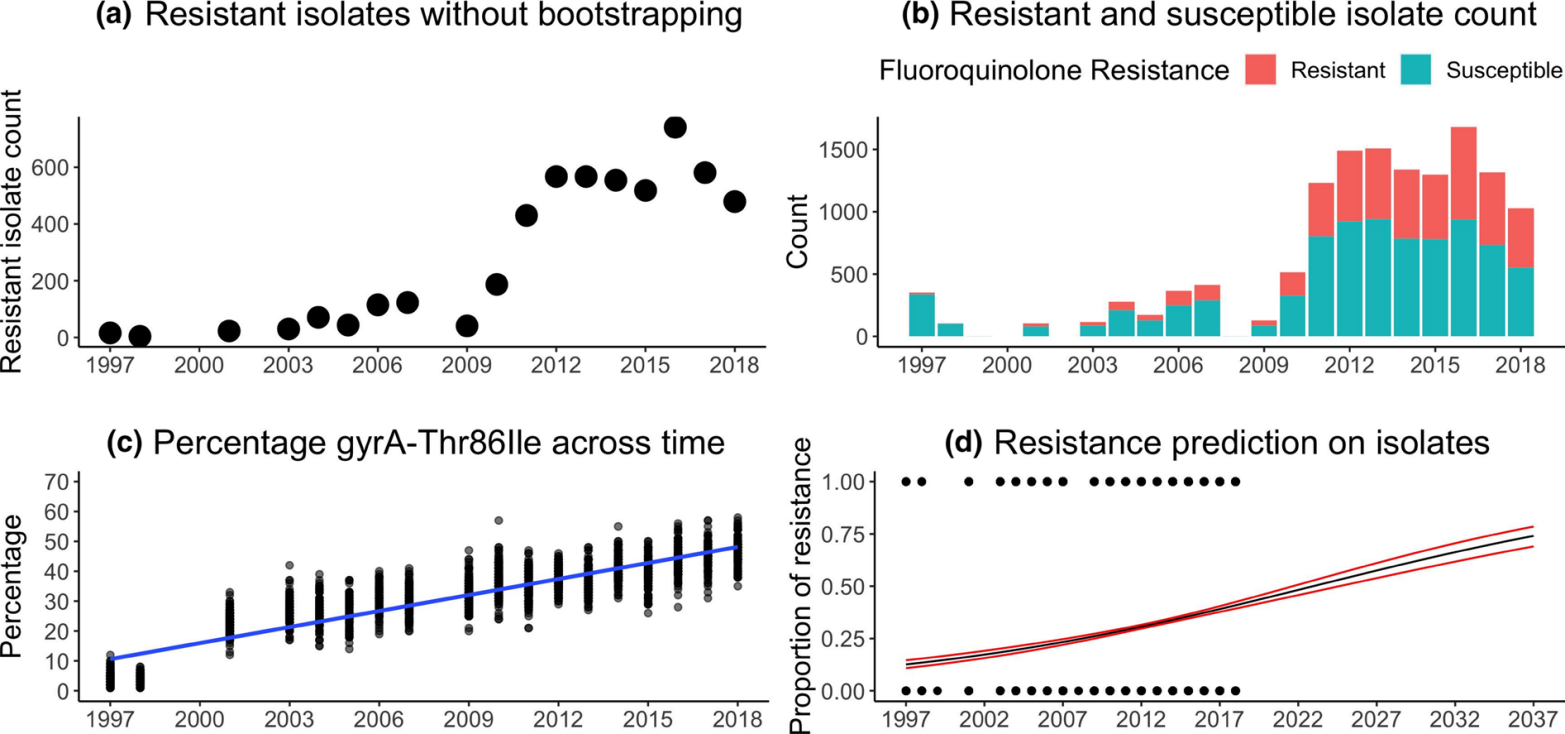
GyrA-Thr86IIe changes across time. (**a**) Isolates counts shown without bootstrapping. (**b**) Resistant and susceptible isolates count across time. (**c**) Percentage of resistant isolates across time with bootstrapping method to give equal sample sizes for each year; 100 isolates randomly picked with 100 iterations. The blue line is a linear regression line (*y*=1.788 × −3560) (d) Prediction of fluoroquinolone resistance isolates by using the generalized model formula in the future years where 1 indicates resistance and 0 indicates susceptible isolates.

In 1998, 5 % of all the isolates were resistant to fluoroquinolones, but by 2018, 45 % of all isolates were resistant to fluoroquinolones (Fig. 1a–c). Despite missing temporal data points (in 1999, 2000, 2002 and 2008), the linear regression analysis suggested that each year the percentage of resistant isolates increased by 1.788 % (linear regression *y*=1.788 × −3560 with R squared: 0.7905 with F statistic: 6761 on 1 and 1791 DF, *P*-value: <0.0001, Fig. 1c). Predictions from this linear regression model showed continued increase in levels of resistance, with ~75 % of all variants predicted to be fully resistant by ~2040 if no effective preventative action is taken (Fig. 1c). A generalized linear model (Fig. 1d) indicated statistical significance of the interaction between the year and groups covariates. The odds of being resistant increased by a log ratio of 1.077 each year [*P*-value<0.001, confidence intervals of 1.066 (2.5 %) and 1.089 (97.5 %)].

### Investigating fluoroquinolone resistance patterns in time

**Fig. 2.**
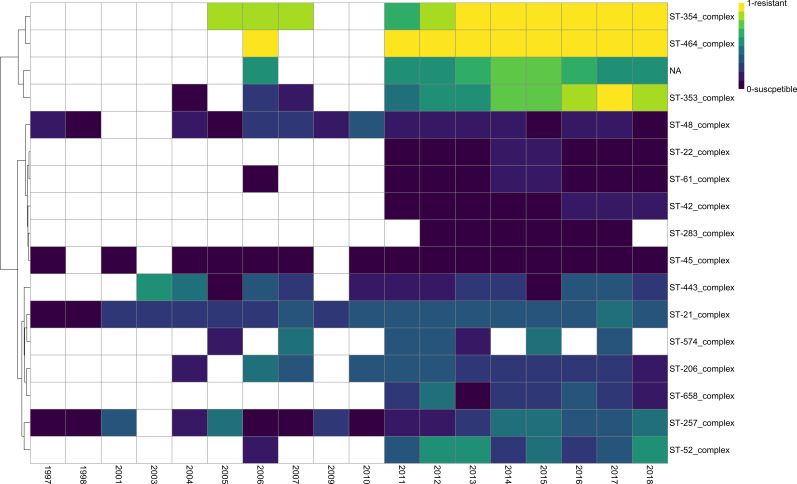
Heatmap showing *C. jejuni* fluoroquinolone resistance by clonal complex across time. Scores were calculated from the resistant isolate count divided by the overall isolate count. Where all isolates were resistant, this gave a value=1 (e.g. 2018 ST-354_complex). White cells indicate there were no data points or omitted due to not passing the quality checks listed below. The top four rows indicate a resistance cluster; rows 5–10 indicate a susceptible cluster and rows 11–17 mixed resistance/susceptible cluster. Only data points containing at least 10 isolates per year, per clonal complex, were used to create this heatmap.

Hierarchical clustering analysis, using all 10,359 isolates and their gyrase 86 positions, revealed 3 specific clusters of fluoroquinolone sensitivity/resistance (Fig. 2). Although some CCs were missing in earlier years, this highlighted the potential emergence of new clonal complexes across time that were not prevalent in early years. Furthermore, we investigated whether the growth promoter ban in 2006 had any influence on the resistance, and for this we used the isolates from 1998. The first cluster, comprising CCs 353, 354 and 464, had a high proportion of resistant isolates. In total, 97.5 % (345 of 354 isolates) of CC464 in this study were resistant. The second cluster, comprising a further six CCs (CCs 48, 22, 61,42, 283 and 45), predominantly included susceptible isolates. Just two CCs, CC45 and CC283, were fully susceptible over the course of the study. The third cluster was composed of a mix of variants where there was no apparent visual pattern that could be recognized, although resistance was generally greater than in the second cluster, with up to 60 % of isolates resistant at a given time.

### Further pattern recognition using trend analysis

**Fig. 3.**
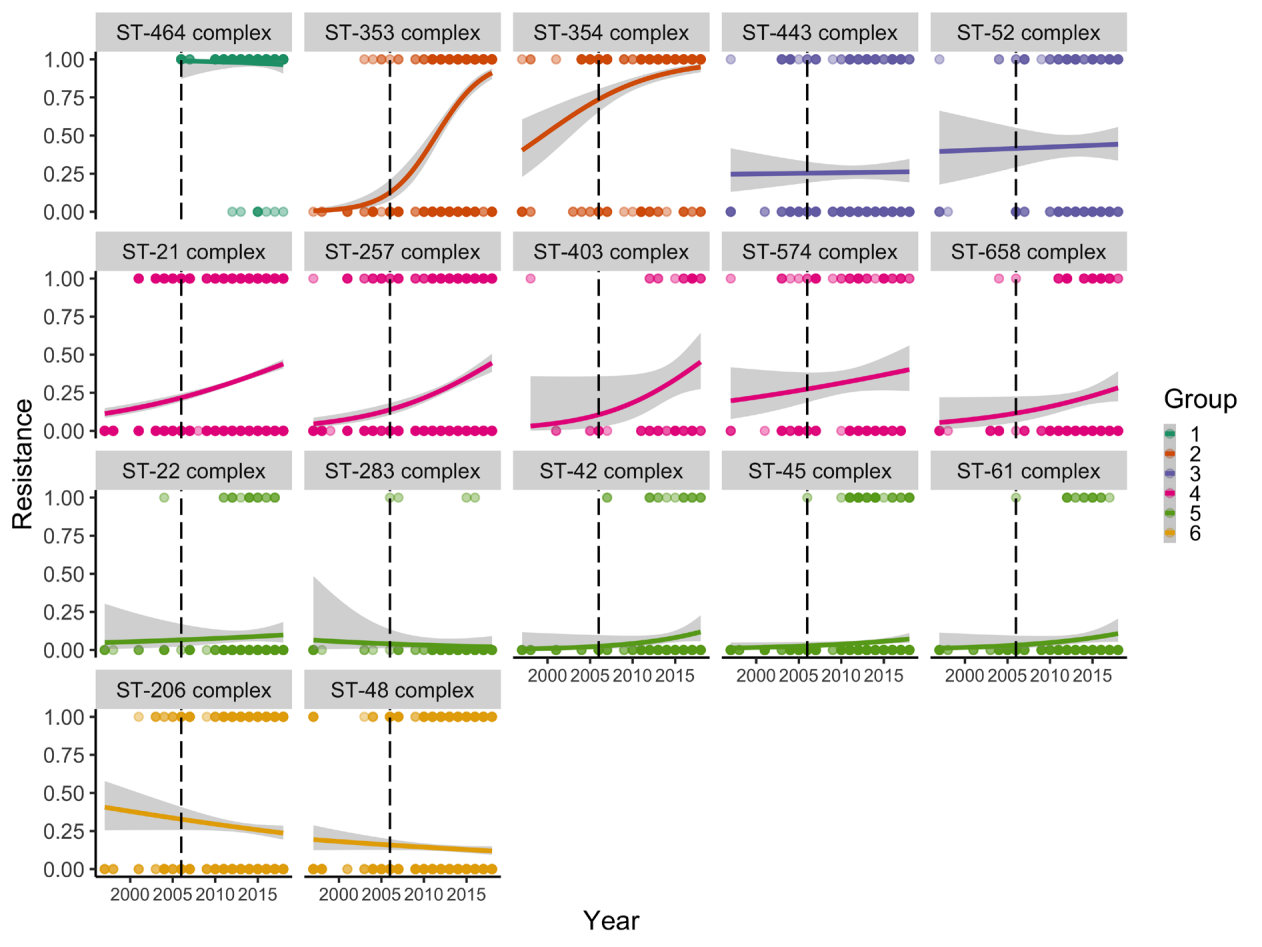
Six patterns in fluoroquinolone resistance changes across clonal complexes. Group 1 illustrates CC464, which predominantly contains resistant isolates. Group 2 contains two clonal complexes, CC353 and CC354, which have had a sharp increase in resistance across time, almost reaching complete resistance in 2018. Group 3 illustrates the outlier cluster, which has no clear trend between resistance and time. Group 4 is a cluster with a weak positive correlation. Group 5 is a cluster that is predominantly susceptible across time. Group 6 is a cluster that has had a slight decrease in resistance. The black dotted line illustrates the 2006 mark where the EU growth promoter ban came into place. Grey shading illustrates the confidence interval. ST indicates clonal complex.

Variants could be assigned to one of three main clusters based on their overall resistance (Fig. 2). However, there could be an underlying pattern or trend across time that is not apparent. Therefore, a generalized linear model was used to investigate and identify the resistance patterns across time for each CC (Fig. 3). The three clusters defined in Fig. 2 could be further differentiated into six patterns; two of high resistance, two of mixed resistance/susceptibility, one showing slight reduced resistance and one of susceptibility over time.

By comparing two generalized linear models, one with three clusters (Fig. 2), with an Akaike information criteria (AIC) value of 10,444, and a second model with six groups (Fig. 3) with an AIC value of 10,311, the second model is a better fit for our data. The analysis further showed that the introduction of a growth promoter ban did not have a significant impact on the emergence and dynamics of resistance. Moreover, since 2006, some CCs, such as CC464, CC353 and CC257, increased in frequency.

**Fig. 4.**
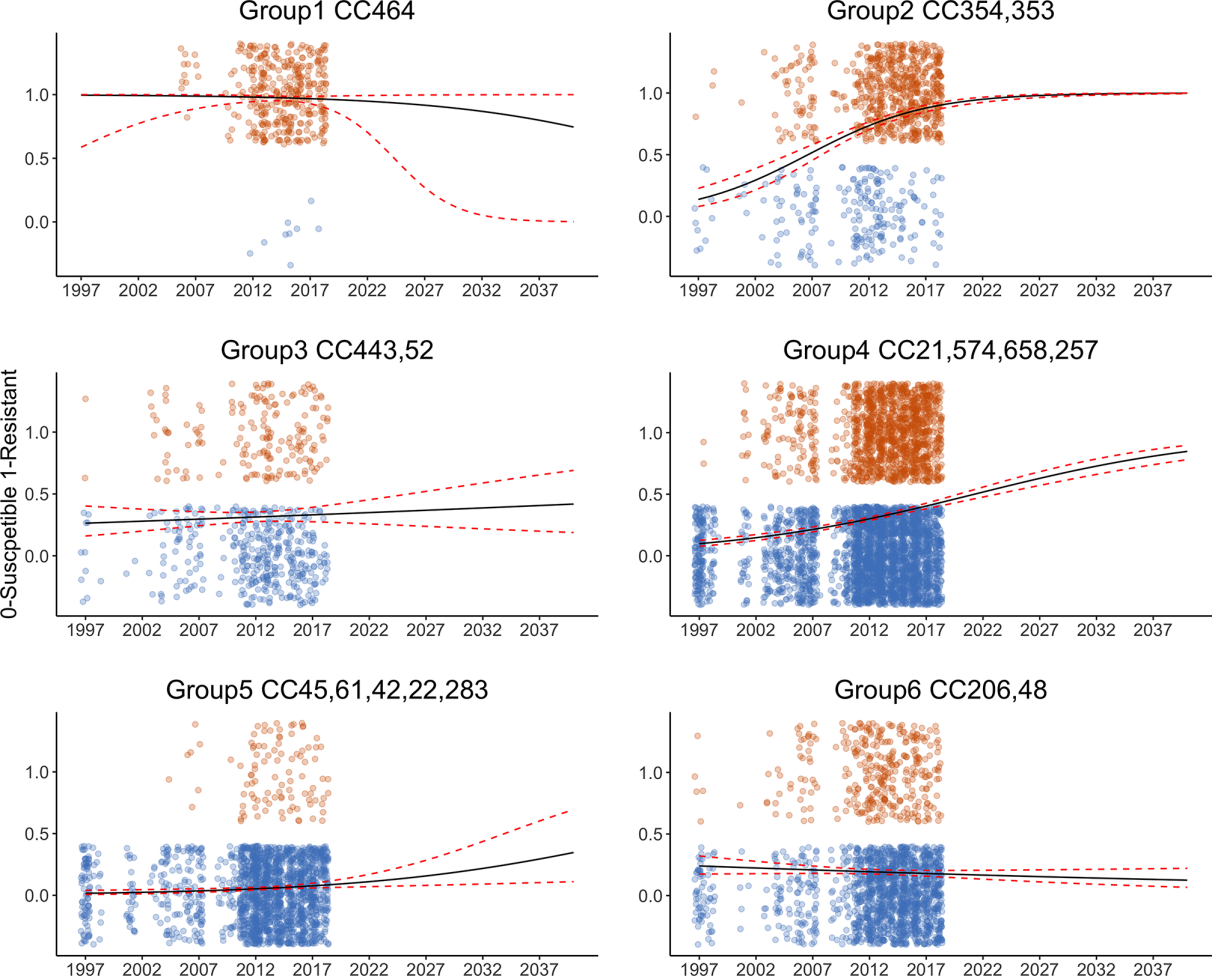
Prediction of fluoroquinolone resistance until 2040. 0 indicates susceptible isolates, 1 indicates the resistant isolates. Jitter function shows the actual number (count) of isolates rather than only having values of 0 and 1, allowing us to see how some groups, such as group 4, have a high number of isolate (counts) compared to group 3, which has a lower number (count). The red lines indicate the confidence intervals. Prediction has been made until 2040.

Using the predict function for generalized linear models (Fig. 4), the odds of group 1 (CC464) being resistant increased by a log ratio of 0.903 each year (*P*-value 0.459; 95 % confidence interval of 0.663 to 1.16). However, future directions of resistance could not be predicted using the model due to the relatively late appearance of isolates from 2011 onwards, with a high proportion of isolates already showing resistance. For group 2 (CC353, CC354) the odds of being resistant increased by a log ratio of 1.210 each year (*P*-value <0.0001; 95 % confidence interval of 1.164 to 1.259). Changes in resistance for group 2 had a sigmoidal shape change from susceptible to resistant, with most isolates predicted to be resistant by 2027.

For group 3 (CC443 and CC52) the odds of being resistant increased by a log ratio of 1.020 each year (*P*-value of 0.429; 95 % confidence interval of 0.977 to 1.059). Due to an almost equal number of isolates across time, the prediction of future resistance trends is difficult, and resistance may potentially stay at the same level in the future. For group 4 (CC574, CC403, CC257, CC21, CC658), the odds of being resistant increased by a log ratio of 1.096 each year (*P*-value <0.0001; 95 % confidence interval 1.078 to 1.114). Group 4 had a tight confidence interval, illustrating a steady increase in fluoroquinolone resistance, but with isolates not predicted to become fully resistant by 2040.

For group 5 (CC283, CC42, CC22, CC61, CC45) the odds of being resistant increased by a log ratio of 1.084 each year (*P*-value of 0.00369; 95 % confidence interval of 1.0299 to 1.15). In terms of prediction, group 5 had a slow increase in resistance but did not follow the steady increase in resistance seen in group 2 or group 4’s steady increase in resistance. For group 6 (CC206, CC48) the odds of being resistant increased by a log ratio of 0.982 each year (*P*-value of 0.143; 95 % confidence interval of 0.958 to 1.007). Group 6 was the only group that indicated a slight decrease in resistance with a tight confidence interval.

In summary, the data indicated that for *C.jejuni* the trend for fluoroquinolone resistance was not uniform for the whole organism but rather exhibited six different patterns related to clonal complexes from 1998 to 2018. These six groups also gave different predictions for fluoroquinolone resistance in the future (Fig. 4)

## Discussion

We investigated the changing temporal patterns of antimicrobial resistance across the CCs of *C. jejuni *. Previous studies of changes in fluoroquinolone resistance in *C. jejuni* have primarily focused on changes at a species level [15, 29], and have not been studied in CCs [23, 30]. While some studies have touched on multiple drug resistance, these patterns have not been thoroughly investigated at the CC level and conclusions have mainly been formed at the species level [31]. A study based in Oxford, UK, indicated that in 1995, 7 % of the overall *Campylobacter* isolates were resistant to fluoroquinolones, but by 2008 this had risen to 37.5 % [32]. Here, we have estimated that if this trend continues, by 2040, up to 75 % of overall *C.jejuni* isolates will have fluoroquinolone resistance.

### Host source attribution

This study examined *C. jejuni* from human disease and did not investigate resistance from other *C. jejuni* sources. However, there have been many studies focused on identifying host source attribution in relation to CC [33, 34, Table S1]. A study focusing on host generalist *C. jejuni* CCs, such as CC45 and CC21, discussed how these CCs have adapted to colonize multiple hosts and estimates indicate that every 1.6 years (CC21) and 1.8 years (CC45) there are transmission events between different hosts, making it very challenging to pinpoint the source of clinical infections [35]. On the other hand, CC1034 and CC702 are commonly associated with wild geese, ducks and environmental waters and are rarely found in patients, indicating that overall these sources make a lower contribution to human infections [36]. CC61 is associated with cattle, and the fact that it has low fluoroquinolone resistance suggests that fluoroquinolone pressure in cattle farming might be lower, compared to poultry farming, where the poultry-associated CC464 and CC354 show high levels of resistance (see source attribution reference in Table S1, [37–39]). Combining host association into the model could be the next step to improve future predictions and identify further patterns for managing resistance.

### Generalized linear model to predict future fluoroquinolone resistance

Aspects of studying fluoroquinolone resistance across time have been considered in previous studies, including a six year study of human disease isolates from 2003 to 2009 that investigated the molecular epidemiology of *Campylobacter* in Oxfordshire, UK, and a review paper that focused on finding trends in fluoroquinolone resistance across time [15, 24]. The six year study of clinical isolates indicated that low susceptibility was found in several CCs (22, 45, 48, 61, 257, 283, 403, 658, 677) and high resistance in other CCs (49, 206, 354, 446, 460, 464, 607) [24]. In comparing these results to those of our study, CC353 was not strongly correlated with being resistant, but Cody *et al*.’s study observed seasonality changes in some CC peaking, such as CC353 being highly present in winter months [24]. Another study focusing on trends in antimicrobial resistance between the UK and USA showed that CC353, CC354 and CC464 have the highest levels of fluoroquinolone resistance in the UK; this finding was corroborated by our results [23]

A different study has found high ciprofloxacin resistance in *C. jejuni *, which has high genetic similarity (CC21 allelic type 1) in central Europe, indicating that this variant is potentially spreading clonally [22]. Another study based in Estonia and Latvia has shown that ST353 and ST5 are the most prevalent CCs found in clinical settings in Estonia, which has high ciprofloxacin resistance [40]. It is known that ST353 and ST5 only differ at one of the seven MLST loci, so the prevalence of these sequence types is not surprising, but it is interesting to note that these two sequence types were found to be high in resistance, whereas in our study, we have discovered CC464 and CC354 to be high in resistance. Our results demonstrate that (i) fluoroquinolone resistance is not uniform across *C. jejuni* CCs and (ii) there is a need for more detailed longitudinal surveillance of antimicrobial resistance at a sub-species level to understand more thoroughly how resistance is evolving.

### Restricted use of antibiotics in the EU and other countries

Other countries in the EU report similar trends in rising quinolone or fluoroquinolone resistance amongst *Campylobacter* isolates over time, although levels of resistance vary across time [41–44]. In Germany, nalidixic acid resistance increased from 8.2 to 26.3 % between 1990 and 2004 [43]. In Italy, high quinolone resistance was significantly associated with *C. jejuni* isolated from food-producing animals despite a ban on fluoroquinolone usage for treatment [41, 42]. In the USA, the Food and Drug Administration (FDA) investigated how the ban on the fluoroquinolone, enrofloxacin, in poultry impacted on levels of resistance [45]. In the USA, fluoroquinolones were banned in 2005 but fluoroquinolone resistance increased from 13 to 25.3 % between 1997 and 2015, which is generally lower than the levels reported in EU countries [46, 47].

These studies indicated more variation within years than between years but concluded that the withdrawal of fluoroquinolones did not contribute to lower resistance levels in *C. jejuni* [45]. In Peru, where there are no current restrictions on antibiotic use, a study compared two periods between 2001–2005 and 2006–2010. Fluoroquinolone resistance increased from 73.1 to 89.9 % between 2001 and 2010, including in the Amazon region of Iquitos, where resistance rose from 24.1 to 48.9 % [48]. In Australia fluoroquinolones have never been licensed for use in the commercial chicken sector [49]. However, a study found that 14.8 % of 108 *C. jejuni* isolates from chickens at slaughter are fluoroquinolone-resistant, despite the absence of fluoroquinolone use for livestock [50]. Amongst these fluoroquinolone-resistant *C. jejuni* isolates, ST7323, ST2083 and ST2343 were highly abundant [50]. ST2343 is part of CC48 and in our study, we found that the overall resistance of this CC decreased, in contrast to trends in the UK to Australia.

Therefore, we found no evidence that the growth promoter ban has had a significant influence on overall fluoroquinolone resistance. Moreover, even in countries, such as Australia, that never licensed antibiotic usage on farms, resistance levels continue to increase.

### Does antibiotic stewardship lead to lower fluoroquinolone resistance levels?

Fluoroquinolone resistance can develop quickly and a study of commercial broiler chickens in the UK compared the level of resistance before, during and after treatment with difloxacin or enrofloxacin clearly shows this effect [51]. This study indicated that once fluoroquinolones were applied, the number of resistant *Campylobacter* isolates increased, and the resistant variants persisted for up to 4 weeks after treatment ended. Many studies have indicated that despite ongoing efforts to improve antibiotic stewardship in both farm and clinical settings, the level of fluoroquinolone resistance has not decreased and instead continues to increase over time [50, 52]. An extensive study analysing the resistome of 39,798 *Campylobacter jejuni* isolates was recently conducted and demonstrated a global trend of increasing tetracycline and fluoroquinolone resistance across time. The chicken-associated CC354, CC573, CC464 and CC446 had the highest levels of resistance [30]. Another *in vivo* study in the USA determined that fluoroquinolone-resistant *Campylobacter* in chickens outcompeted fluoroquinolone-susceptible variants, illustrating that resistant isolates are biologically more fit in the chicken host [18].

The fact that resistant isolates have a fitness benefit raises the question of what underlying biological mechanisms could be selected for with this mutation in some variants but not others. The resistant clusters, CC464, CC354 and CC353, are known to be more poultry related, whereas CC42 and CC61, which are low in resistance, are known to be cattle related (Table S1). In addition, studies suggest that resistant isolates are associated with higher virulence and survival phenotypes, raising questions about why, although the regulations have changed, we do not see decreases in resistance in some of the variants [23]. A recent study indicated that the acquisition of fluoroquinolone resistance leads to an increase in biofilm formation, which leads to an increased ability to survive in the external environment, and also higher pathogenicity in human infection [53]

Here we examined fluoroquinolone resistance, but the isolates could be resistant to other antibiotics deriving from different mechanisms. A study compared UK and USA isolates and their different antibiotics markers revealed that CC354 and CC464 are high in both fluoroquinolone and tetracycline resistance, while some CCs, such as CC206, are only high in tetracycline resistance, showing different patterns from those in our study [23]. Further investigating the differences between each country’s antibiotic regulations and farming and cultural features, including the food chain stages, could provide additional insights into why CCs differ.

### Conclusion

Overall, *C. jejuni* is showing an increase in fluoroquinolone resistance over time in the UK, leading to a prediction of 75 % overall resistance by 2040. Our clustering analysis has shown that resistance levels vary by clonal complex, with CC353, CC354 and CC464 being the most resistant, and predicted to become almost 100 % resistant to fluoroquinolones by 2040. Our results indicate that consideration of species variant is essential for elucidating the epidemiology and emergence of antimicrobial resistance for this diverse organism.

## Supplementary Data

Supplementary material 1
